# Vemurafenib plus cobimetinib in unresectable stage IIIc or stage IV melanoma: response monitoring and resistance prediction with positron emission tomography and tumor characteristics (REPOSIT): study protocol of a phase II, open-label, multicenter study

**DOI:** 10.1186/s12885-017-3626-5

**Published:** 2017-09-15

**Authors:** Bernies van der Hiel, John B.A.G. Haanen, Marcel P.M. Stokkel, Daniel S. Peeper, Connie R. Jimenez, Jos H. Beijnen, Bart A. van de Wiel, Ronald Boellaard, Alfons J.M. van den Eertwegh, REPOSIT study group

**Affiliations:** 1 Department of Nuclear Medicine, Netherlands Cancer Institute – Antoni van Leeuwenhoek Hospital, PO Box 90203, 1006 BE Amsterdam, The Netherlands; 2 Department of Medical Oncology, Netherlands Cancer Institute – Antoni van Leeuwenhoek Hospital, Amsterdam, the Netherlands; 3 Department of Molecular Oncology, The Netherlands Cancer Institute, Amsterdam, The Netherlands; 4 Oncoproteomics Laboratory, Department of Medical Oncology, VU University Medical Center, Amsterdam, the Netherlands; 5 Department of Pharmacy and Pharmacology, The Netherlands Cancer Institute – Antoni van Leeuwenhoek Hospital, Amsterdam, The Netherlands; 6 Department of Pathology, The Netherlands Cancer Institute – Antoni van Leeuwenhoek Hospital, Amsterdam, the Netherlands; 7 Department of Nuclear Medicine and Molecular Imaging, University Medical Center Groningen, Groningen, The Netherlands; 8 Department of Medical Oncology, VU University Medical Center, Amsterdam, the Netherlands

**Keywords:** Stage IIIc/IV melanoma, Resistance, BRAF inhibitor, MEK inhibitor, ^18^F-FDG, ^18^F-FLT, PET/CT

## Abstract

**Background:**

In patients with BRAFV600 mutated unresectable stage IIIc or metastatic melanoma, molecular targeted therapy with combined BRAF/MEK-inhibitor vemurafenib plus cobimetinib has shown a significantly improved progression-free survival and overall survival compared to treatment with vemurafenib alone. Nevertheless, the majority of BRAFV600 mutation-positive melanoma patients will eventually develop resistance to treatment.

Molecular imaging with ^18^F-Fluorodeoxyglucose (^18^F-FDG) PET has been used to monitor response to vemurafenib in some BRAFV600 mutated metastatic melanoma patients, showing a rapid decline of ^18^F-FDG uptake within 2 weeks following treatment. Furthermore, preliminary results suggest that metabolic alterations might predict the development of resistance to treatment. ^18^F-Fluoro-3′-deoxy-3’L-fluorothymidine (^18^F-FLT), a PET-tracer visualizing proliferation, might be more suitable to predict response or resistance to therapy than ^18^F-FDG.

**Methods:**

This phase II, open-label, multicenter study evaluates whether metabolic response to treatment with vemurafenib plus cobimetinib in the first 7 weeks as assessed by 18F-FDG/18F-FLT PET can predict progression-free survival and whether early changes in 18F-FDG/18F-FLT can be used for early detection of treatment response compared to standard response assessment with RECISTv1.1 ceCT at 7 weeks.

Ninety patients with BRAFV600E/K mutated unresectable stage IIIc/IV melanoma will be included. Prior to and during treatment all patients will undergo ^18^F-FDG PET/CT and in 25 patients additional ^18^F-FLT PET/CT is performed. Histopathological tumor characterization is assessed in a subset of 40 patients to unravel mechanisms of resistance. Furthermore, in all patients, blood samples are taken for pharmacokinetic analysis of vemurafenib/cobimetinib. Outcomes are correlated with PET/CT-imaging and therapy response.

**Discussion:**

The results of this study will help in linking PET measured metabolic alterations induced by targeted therapy of BRAFV600 mutated melanoma to molecular changes within the tumor. We will be able to correlate both ^18^F-FDG and ^18^F-FLT PET to outcome and decide on the best modality to predict long-term remissions to combined BRAF/MEK-inhibitors. Results coming from this study may help in identifying responders from non-responders early after the initiation of therapy and reveal early development of resistance to vemurafenib/cobimetinib. Furthermore, we believe that the results can be fundamental for further optimizing individual patient treatment.

**Trial registration:**

Clinicaltrials.gov identifier: NCT02414750. Registered 10 April 2015, retrospectively registered.

## Background

Cutaneous malignant melanoma develops from melanocytes, which can either occur de novo or from a pre-existing lesion such as an acquired, congenital or dysplastic nevus. Worldwide, the highest incidence in melanoma is in Australia and New Zealand, but also in Europe and North America incidence is rising [1]. In 2013, more than five thousand (5489) newly diagnosed patients with skin melanoma were treated in the Netherlands [2]. Fifteen to twenty percent of these patients develop stage IV melanoma and in 2013 about 800 patients died from melanoma.

For many years, the only available systemic therapy was the chemotherapeutic agent dacarbazine, resulting in poor response rates (around 5–20%) and no significant survival benefit. This changed in 2011 with the introduction of the immune checkpoint inhibitor ipilimumab and inhibitor of the MAP kinase pathway vemurafenib, anti-melanoma agents that improved progression free and overall survival of patients with advanced melanoma [3, 4]. After the introduction of these revolutionary therapies the BRAF inhibitor dabrafenib, MEK-inhibitors trametinib and cobimetinib, and the anti-PD1 antibodies nivolumab and pembrolizumab were approved for the treatment of metastatic melanoma. Despite the introduction of these practice-changing drugs in the treatment of metastatic melanoma a number of critical issues remains to be settled. First, what is the optimal sequence of immunotherapy and targeted therapy? Second, can we predict and overcome resistance against these agents?

Targeted cancer therapies include drugs that interfere with oncogenic molecules involved in cancer cell growth and survival. The Mitogen Activated Protein Kinase (MAPK) pathway is a chain of proteins that in normal cells regulates cell division, differentiation and secretion through relaying extracellular signals from cell membrane to nucleus via a cascade of phosphorylation events. Dysregulation of the MAPK pathway plays a major role in malignant transformation of cells. The BRAF kinase protein is one of the proteins responsible for regulating the MAPK pathway. In many human cancers, dysregulation of the MAPK pathway is caused by a mutation in the BRAF gene. In melanoma, BRAF mutations account for roughly 40–50% of all cases and is most common in so-called non-chronic sun damage melanomas [5, 6]. The most frequent BRAF mutation is the BRAFV600E mutation, situated in the kinase domain of the protein, in which a Glutamic acid is substituted for Valine at residue 600 in ATP binding pocket [6, 7]. This BRAFV600 mutation represents a unique target for cancer therapy and has led to the development of several specific BRAF inhibitors, amongst others vemurafenib. In melanoma patients, this oral inhibitor of mutated BRAF protein only works in the presence of a BRAFV600E or V600 K mutation; melanoma cells without this mutation are not inhibited.

Results from the randomized controlled phase III trial (BRIM3) comparing vemurafenib to standard dacarbazine chemotherapy in newly diagnosed patients with BRAFV600E mutation positive metastatic melanoma revealed a median PFS of 6.9 months for vemurafenib and 1.6 months for dacarbazine (HR 0.26; 95% CI, 0.20–0.33; *p* < 0.0001). An objective response rate of 48.8% was found in the vemurafenib group, compared to a response rate of 5% with dacarbazine. In addition, this study showed a significant survival benefit in favor of vemurafenib [4, 8].

These improved overall and progression-free survival have led to approval of vemurafenib by the Food and Drug Administration (FDA) on August 17, 2011, as a first line treatment in patients with BRAF-mutated unresectable or metastatic melanoma. On May 29, 2013, also BRAF inhibitor dabrafenib was approved by the FDA, based on demonstration of improved PFS in a phase III multi-center, international, open-label, randomized controlled trial [9].

With these highly encouraging data, however, new problems appeared. The biggest dilemma is the relative short duration of BRAF inhibitor-induced responses due to the development of resistance to these drugs, which eventually occurs in all treated patients. However, while these mechanisms appear to be much more pleiotropic than expected, on an individual basis we are unable to forecast when resistance will set in. Another problem is that even though the majority of patients benefit from the treatment, still a number of patients do not benefit at all as progression of the disease continues after the initiation of therapy. At this point it is not possible to select patients who will benefit from this therapy in advance or early after initiation of therapy.

The major resistance mechanism to BRAF inhibition is reactivation of the MAPK pathway, either due to amplification of the mutated BRAF gene, occurrence of splice variants of mutated BRAF, activating MEK mutations, activating RAS mutations or epigenetic alterations leading to activation of the MAPK or PI3K pathway [10, 11]. This has led to the development of MEK-targeted therapies that target this pathway further downstream. Although in a phase III study a MEK inhibitor has demonstrated an OS benefit over chemotherapeutic drugs in patients with advanced melanoma containing a BRAFV600E mutation [12], the clinical usefulness of MEK inhibitors as single agent in the treatment of advanced melanoma is uncertain. This is mainly because the response rates with MEK inhibitors do not reach those of selective BRAF inhibitors [8, 9].

### Combined BRAF and MEK inhibition

Based on the mechanisms of resistance to BRAF inhibition combining BRAF inhibitors with MEK inhibitors was investigated. In three randomized phase III trials combined BRAF- and MEK inhibition out-performed BRAF inhibitor monotherapy.

In the COMBI-d study patients with advanced melanoma were randomly assigned to receive dabrafenib plus trametinib or dabrafenib plus placebo as a first-line treatment. Median PFS provided only a minor advantage compared to monotherapy (9.3 vs. 8.8 months; *p* = 0.03). In addition, combined BRAF and MEK inhibition resulted in a 25% relative reduction in the risk of disease progression compared with dabrafenib alone, as well as a significant higher response rate (67 vs. 51%; *p* = 0.002) [13].

In May 2015, Long et al. published follow-up data on median OS, which was 25.1 months for patients in the dabrafenib plus trametinib group versus 18.7 months for patients in the dabrafenib-only group (HR 0.71, 95% CI 0.55–0.92; *p* = 0.0107). Based on 301 events, median PFS was 11.0 months (95% CI 8·0–13·9) in the dabrafenib and trametinib group and 8.8 months (5.9–9.3) in the dabrafenib only group (HR 0.67, 95% CI 0.53–0.84; *p* = 0.0004) [14].

In the COMBI-v study 704 patients with BRAF mutated metastatic melanoma were randomly assigned to receive either a combination of dabrafenib plus trametinib or vemurafenib alone as a monotherapy. The primary endpoint was OS, the study was stopped early because at the interim analysis OS was longer in patients treated with the combination compared to patients given the monotherapy (median OS not reached vs. 17.2 months), as well as higher PFS (11.4 vs 7.3 months; HR 0.56, 95% CI 0.46–0.69; *p* < 0.001), duration of response (13.8 vs. 7.5 months) and response rate (64 vs. 51%; *p* < 0.001) [15].

In the 3rd phase III study, coBRIM, untreated BRAF-mutant patients were randomized to receive BRAF inhibitor vemurafenib plus MEK inhibitor cobimetinib or vemurafenib plus placebo: combination therapy resulted in improved PFS (9.9 vs. 6.2 months; HR 0.51, 95% CI 0.39–0.68; *p* < 0.001), 9-month OS rate (81.1 vs. 72.5%) and response rate (68 vs. 45%; *p* < 0.001) [16]. In an updated report presented at the SMR 2015 an improved PFS (12.3 vs 7.2 months, *p* < 0.001) and overall survival (22.3 vs 17.4 months, *p* = 0.005) was shown in favour of the combined vemurafenib plus cobimetinib arm [17].

Based on these clinical trials revealing the clinical benefit of the combination the FDA approved in November 2015 vemurafenib plus cobimetinib as well as dabrafenib plus trametinib for the treatment of patients with unresectable or metastatic melanoma with BRAF V600E or V600K mutations.

### Positron emission tomography

Positron Emission Tomography / Computed Tomography (PET/CT) is a nuclear medicine technique that produces a three-dimensional image of functional processes in the body combined with anatomical mapping by means of CT. Depending on the type of tracer, a diverse of molecular processes can be visualized such as glucose metabolism, proliferation or hypoxia.

^18^F-Fluorodeoxyglucose (^18^F-FDG) is the radiotracer mostly used in clinical application of PET. ^18^F-FDG is a radiolabelled glucose analogue that replicates the glucose metabolism. Unlike glucose, ^18^F-FDG is phosphorylated, causing a stop to further metabolism and, consequently, trapped in the cell. Since the glucose metabolism is higher in tumor cells than in surrounding normal cells, malignant lesions show enhanced ^18^F-FDG uptake on a PET-scan. ^18^F-FDG PET/CT has been widely studied in staging, restaging and response assessment in almost any type of malignant tumors. In the guidelines of Non Small Cell Lung cancer, ^18^F-FDG PET/CT has become one of the most important tools in staging mediastinal lymph node involvement. Several meta-analyses revealed a higher sensitivity and specificity with ^18^F-FDG PET compared to diagnostic CT [18–21].

Generally, the utility of ^18^F-FDG PET imaging in melanoma patients depends on the stage of the tumor. In Stage I and II melanoma, ^18^F-FDG PET appears to have no additional value since micrometastases are missed because ^18^F-FDG PET cannot detect small tumor volumes [22, 23]. Even in patients with proven tumor positive sentinel lymph nodes, staging with ^18^F-FDG PET results in a high percentage of false positives and false negatives [24, 25]. On the contrary, ^18^F-FDG PET does seem to be valuable in Stage III and IV melanoma, especially in detecting distant metastases and recurrence as well as in monitoring therapy response. At the NKI, Aukema et al. showed in 70 patients with palpable lymph node metastases an 87% sensitivity and 98% specificity in the detection of other metastases [26]. In this study, ^18^F-FDG PET/CT led to a change in treatment in 37% of patients. Though routinely CT is performed for detecting distant metastases in stage III melanoma, ^18^F-FDG PET/CT has shown superior performance compared to CT alone [27, 28]. Sensitivity and specificity of ^18^F-FDG PET/CT was 86 and 91% respectively whereas for CT alone a sensitivity of 65% and specificity of 78% was reported. For the detection of metastatic disease in the brain, liver and bone, MRI is superior to ^18^F-FDG PET/CT [29]. Therefore, a combination of ^18^F-FDG PET/CT and MRI is the most efficient manner to perform whole body surveillance in high-risk stage III and IV melanoma.

### Response monitoring with PET

For monitoring therapy response in unresectable stage IIIc or metastatic melanoma, contrast enhanced CT (ceCT) is the current standard imaging tool. Unfortunately, reduction in tumor size cannot be assessed within days after the initiation of therapy and anatomic size does not provide information about the development of therapy response or resistance at a molecular level. It has been clearly demonstrated that alterations in metabolism occur earlier than anatomical size reduction after the initiation of therapy. PET imaging is a sensitive method to detect alterations in cell metabolism, even within 15 days after the start of therapy [30]. By detecting these metabolic alterations, it is suggested that responders can be distinguished from non-responders at an earlier phase compared with anatomical imaging with ceCT [31]. This way, unnecessary expensive treatment with combined BRAF/MEK inhibition and its side effects may be prevented in patients who will not benefit from this therapy.

^18^F-FDG PET in monitoring response assessment has been investigated in many tumors, but has not been studied systematically in metastatic melanoma patients, due to the lack of an efficacious treatment. With the promising results in OS and PFS in patients treated with combined BRAF/MEK inhibition, response monitoring becomes a topic of high interest. Sondergaard et al. studied the effect of vemurafenib in different in-vitro cell lines, all with a BRAFV600 mutation [32]. They demonstrated a different sensitivity to PLX4032 in all ten cell lines, three of these cell lines even showing resistance. Inhibition of ^18^F-FDG uptake in the cell lines was more profound in the sensitive cell lines than in the resistant cell lines, suggesting that ^18^F-FDG PET can be used as a non-invasive imaging technique to distinguish between sensitive and resistant tumors.

In a dose-escalation study of vemurafenib, ^18^F-FDG PET/CT was performed in a selective group of patients at baseline and on day 15 of the first 4 weeks of therapy [30, 33]. ^18^F-FDG PET/CT showed a decrease in uptake in all patients, corresponding with therapy response. Furthermore, preliminary results in literature suggest a positive association between the level of decline in metabolic activity in the first 2 weeks after the initiation of therapy and PFS and OS [31]. In a preclinical study it was demonstrated that in mutant melanomas treated with vemurafenib and the MEK inhibitor cobimetinib (GDC-0973), metabolic imaging with ^18^F-FDG PET appeared to be a good biomarker of both early response assessment and acquired resistance [34]. If clinically validated, one could switch metabolic non-responders to early additional therapy, guided by phosphoprotein and/or DNA mutation profiles, with other targeted therapies (e.g. ERK-inhibitors or PI3-kinase inhibitors) or change to immunotherapy.

### ^18^F-FDG versus ^18^F-FLT PET

As mentioned before, BRAF inhibition interferes in the MAPK pathway through inhibition of oncogenic mutated BRAFV600. As a consequence, cell proliferation is altered. ^18^F-fluoro-3′-deoxy-3′-L-fluorothymidine (^18^F-FLT) is a tracer developed for PET, which can visualize cell proliferation in vivo. In the S phase, ^18^F-FLT is phosphorylated to 3′-fluorothymidine monophosphate by thymidine kinase 1, after which it is trapped in the cell due to its negative charge. Thus, ^18^F-FLT accumulates in proliferating tissue and the uptake is reduced in cells that are in the growth-arrested phase [35]. Compared to ^18^F-FDG, ^18^F-FLT has a lower overall uptake and higher background activity in liver and bone marrow [36]. The use of ^18^F-FLT PET in oncology should be considered a powerful addition to ^18^F-FDG PET, providing additional information that could be useful in predicting prognosis, planning treatment and monitoring response [37]. Increased DNA synthesis is potentially more tumor-specific than high glucose metabolism and may correspond more directly with tumor aggressiveness and response to therapy. Therefore, it is likely to assume that ^18^F-FLT could be of additional value in stage III/IV melanoma.

Only four studies have investigated in vivo the value of ^18^F-FLT in melanoma compared to ^18^F-FDG, and only one of these response to anti-cancer vaccination was studied [38]. In two studies, ^18^F-FLT appeared to be a good tracer in the evaluation of response to therapy with BRAFV600 mutation in mice [39, 40]. In addition, in the study by Solit et al., ^18^F-FLT seemed more sensitive to treatment response compared to ^18^F-FDG (mean decline of uptake of 43% in ^18^F-FLT versus 16% in ^18^F-FDG). In both studies only a MEK inhibitor was used. Response assessment of BRAFV600E inhibitors in preclinical models of colorectal cancer showed also that ^18^F-FLT is a sensitive predictor, perhaps even better than ^18^F-FDG [41]. Geven et al. evaluated these tracers for response assessment of a BRAFV600E inhibitor in mice with melanoma xenografts. They concluded that only ^18^F-FDG PET is useful as an imaging biomarker for the evaluation of early response [42]. No studies are available in which a BRAF inhibitor plus MEK inhibitor is used to compare ^18^F-FLT to ^18^F-FDG in response assessment.

### Genomics and phosphoproteomics in melanoma

The underlying mechanism of resistance to BRAFV600 inhibitors that have been discovered to date lies in alterations in, and parallel to, the MAPK pathway. However, although this compound, or drugs inhibiting other components within this pathway, initially reduces tumor burden dramatically, eventually virtually all melanomas become resistant and patients succumb to the disease. Drug resistance has therefore become subject to intense study, which has led to the identification of a plethora of mechanisms. For example, several mutations located in the MAPK pathway have been identified, including the C121S mutation in MEK1, Q61K in NRAS, K117 N in KRAS and a gatekeeper mutation T529 in BRAFV600 ([10, 11] and references therein). In addition, long-term treatment with BRAF inhibitor has been shown to induce switching between RAF isoforms, amplification of BRAFV600 or expression of an alternative 61-kDa RAF splice variant lacking the RAS-binding domain. Other mechanisms, not involving the MAPK-pathway, have been found as well, like upregulation of IGF-1R, EGFR, PDGFRβ, FOXP3 or FGFR3 signaling [43–45]. Overexpression of COT, Cyclin D1 and amplification of MET and CTNNB1 can confer resistance to vemurafenib, too [10, 11]. Lastly, the stroma can play a decisive role in acquiring resistance, as it was found that upregulation of HGF by the surrounding stroma occurs during resistance [46, 47].

Phosphoproteomics allows for unbiased phosphoprotein profiling and thereby may yield novel insights into aberrantly activated signaling pathways in cancer cells and tissues in situ. Therefore, phosphoproteomics may identify the above-mentioned mechanisms including alterations in MAPK-pathway activity and may uncover additional, novel mechanisms. Recent phosphoproteomics studies in melanoma cell lines have investigated basal and kinase inhibitor driven adaptive signaling, and have provided insight into the molecular nature of the (combinatorial) response and resistance [48–52].

### Pharmacokinetics

As mentioned earlier, despite proven efficacy of BRAF inhibitors in BRAFV600 mutation-positive metastatic melanoma, responses remain temporary.

Failure arises most likely from induction of resistance to these agents but pharmacokinetic variability may contribute as well. The ease of oral administration enables patients to get their drug regimen in an outpatient setting, which is more patient friendly. However, oral administration also entails the possibility of variable drug exposure due to patient non-compliance, drug interactions with co-medication and variability in oral drug availability.

The relation between treatment outcome (adverse effects and/or treatment failure) and dose has been described in clinical studies. However plasma concentrations have been described only in a single phase I clinical trial on day one and day 15. These results showed large interpatient variability [33]. Pharmacokinetic variability (both interpatient and intrapatient) may be an important factor for treatment outcome, but more data should be collected.

## Methods/design

### Trial design

The Reposit-study is a national multi-center open-label single arm explorative phase II clinical study.

### Patient population

BRAFV600E or BRAFV600K mutation-positive patients with unresectable locally advanced or metastatic melanoma will be included in the trial. Inclusion and exclusion criteria are listed in detail in Table 1, written informed consent is required for inclusion.

**Table 1:** Inclusion and exclusion criteria

Inclusion criteria	Exclusion criteria
***Disease-Specific Inclusion Criteria:*** • Histologically confirmed melanoma, either unresectable stage IIIc or stage IV metastatic melanoma, as defined by AJCC 7th edition. • Naïve to treatment for locally advanced unresectable or metastatic disease (i.e., no prior systemic anti-cancer therapy for advanced disease; stage IIIc and IV). Prior immunotherapy (including ipilimumab) is allowed. • Documentation of BRAF^V600E^ or BRAF^V600K^ mutation-positive status in melanoma tumor tissue. • Measurable disease per RECIST v1.1, which are accessible to biopsies. • Biopsy lesion is within scan reach of contrast enhanced CT and PET/CT. • ECOG performance status of 0 or 1. • Consent to undergo tumor biopsies of accessible lesions.	***Cancer-Related Exclusion Criteria:*** • History of prior RAF or MEK pathway inhibitor treatment. • Palliative radiotherapy, major surgery or traumatic injury within 14 days prior to the first dose of study treatment. • Active malignancy other than melanoma that could potentially interfere with the interpretation of efficacy measures.
	***Exclusion Criteria Based on Organ Function:*** • History of or evidence of retinal pathology on ophthalmologic examination that is considered a risk factor for neurosensory retinal detachment, retinal vascular occlusion, or neovascular macular degeneration. • History of clinically significant cardiac dysfunction, including current unstable angina, symptomatic congestive heart failure (NYHA classII), history of congenital long QT syndrome or mean QTcF >450 msec at baseline or uncorrectable abnormalities in serum electrolytes, uncontrolled hypertension ≥ Grade 2, left ventricular ejection fraction (LVEF) below institutional lower limit of normal (LLN) or below 50%. • Patients with active CNS lesions, except when all known CNS lesions have been treated with stereotactic therapy or surgery, AND there has been no evidence of clinical and radiographic disease progression in the CNS for ≥3 weeks after radiotherapy or surgery.
***General Inclusion Criteria:*** • Male or female patient aged ≥18 years. • Able to participate and willing to give written informed consent. • Life expectancy ≥12 weeks. • Adequate hematologic, hepatic and renal function. • Use of adequate contraception during the course of this study and for at least 6 months after completion of study therapy. • Negative serum pregnancy test in women of childbearing potential. • Absence of any psychological, familial, sociological, or geographical condition that potentially hampers compliance with the study protocol and follow-up after treatment discontinuation schedule.	
	***General Exclusion Criteria:*** • Current severe, uncontrolled systemic disease. • History of malabsorption or other condition that would interfere with absorption of study drugs. • Pregnant, lactating, or breast-feeding. • Unwillingness or inability to comply with study and follow-up procedures. • St. John’s wort or hyperforin (potent cytochrome P450 CYP3A4 enzyme inducer) and grapefruit juice (potent cytochrome P450 CYP3A4 enzyme inhibitor) are prohibited at least 7 days prior to initiation of and during treatment.

### Study objectives

The primary aims of this study are to evaluate whether changes in ^18^F-FDG and/or ^18^F-FLT PET in the first 7 weeks can predict progression-free survival and whether early changes (at 2 weeks) in ^18^F-FDG and/or ^18^F-FLT PET can be used for early detection of response to treatment with combined BRAF/MEK inhibitor vemurafenib plus cobimetinib compared to standard response assessment with ceCT according to RECIST v1.1 at 7 weeks.

Several secondary aims are defined in this study. The level of target inhibition is studied to whether it can predict the type (CR/PR, SD or PD) and duration of response. Molecular mechanisms of resistance will be elucidated by integrating global protein phosphorylation, genomic and cancer mutation data. The correlation between PET imaging and several key tumor characteristics at different levels is studied through a) measurements of BRAF signaling activity by immunohistochemistry (including pERK), b) analyzing DNA mutation spectrum by next-generation sequencing, c) analyzing gene expression patterns by microarray analysis and d) analyzing protein phosphorylation patterns by mass spectrometry-based phosphoproteomics. We will investigate whether differences in global phosphoprotein (including markers of BRAF pathway activity) and mutation profiles occur between sensitive and matched resistant tumor lesions and whether these profiles can predict benefit from vemurafenib/cobimetinib and resistance. Furthermore, we will investigate whether differences in protein phosphorylation patterns can be identified between pre and post treatment tumor biopsies, and whether the pre-treatment patterns would bare predictive power for response to treatment. The correlation between the pharmacokinetics of vemurafenib/cobimetinib and PET imaging is studied as well as the correlation between pharmacokinetics and therapy response. Finally, various quantitative measures of radiotracer uptake from baseline upon PET imaging are studied for predicting the type (CR/PR, SD or PD) of response, progression-free survival and overall survival.

### Study endpoints

Two primary endpoints are defined: (1) Changes in Standardized Uptake Value (SUV) of ^18^F-FDG and ^18^F-FLT PET, assessed on a continuous scale and categorized using PERCIST criteria, (2) Progression-free survival.

The secondary endpoints are as follows:

- Diagnostic accuracy of metabolic tracer uptake on PET in responders and non-responders.
- Glycolytic Index, Metabolic Tumor Volume and % Injected Dose of ^18^F-FDG/FLT on PET.
- Immunohistochemical analysis of tumor tissue in responders and non-responders.
- Changes of DNA in tumor tissue as measured by DNA deep sequencing analysis.
- Changes of RNA in tumor tissue as measured by RNA expression analysis.
- Changes of phosphoproteomic profiles in tumor tissue measured by nano-liquid chromatography coupled to tandem mass spectrometry (nanoLC-MS/MS).
- Changes in vemurafenib and cobimetinib drug concentrations in plasma as measured by a validated Liquid Chromotography tandem Mass Spectrometry assay.
- Overall Survival.
- ECOG Performance status.

### Overview of study design

This national multicenter prospective clinical phase II study will include approximately 90 BRAFV600E or BRAFV600 K mutation-positive patients with unresectable locally advanced or metastatic melanoma. All patients included in the study will be treated with vemurafenib 960 mg BID plus cobimetinib 60 mg QD.

The study is composed of 4 periods:

- *Screening Period* during which screening procedures will take place in order to meet the inclusion criteria,
- *Baseline Period* during which baseline study procedures will take place (^18^F-FDG/^18^F-FLT PET, Pharmacokinetic (PK) blood samples, tissue sampling),
- *Treatment Period* during which patients are treated with vemurafenib plus cobimetinib and during which study procedures will take place,
- *Follow-up Period* during which late side effects are monitored after end of treatment.

During the Screening Period, Baseline Period and Treatment Period, patients are considered ‘on study’; during Follow-up Period, patients are considered ‘off study’.

Laboratory assessments, physical examination, dermatologic examination, ophthalmology examination and cardiac evaluation will take place during the study to monitor safety and side effects of vemurafenib and cobimetinib.

All 90 patients will undergo ^18^F-FDG PET prior to treatment, 2 weeks after the initiation of therapy, at the end of the seventh week and at progression to compare to regular ceCT.

Additional PET with ^18^F-FLT will be performed at baseline, 2 weeks and at progression, PK blood samples will be collected for drug level monitoring of vemurafenib/cobimetinib and biopsies are taken for histopathological tumor characterization.

To answer all main and secondary objectives, ^18^F-FLT PET and tissue samples are only needed in a subset of patients. Therefore, this cohort is divided into three substudies:

- Substudy 1: 25 patients undergoing ^18^F-FDG PET, PK blood sampling, tumor
  biopsies and ^18^F-FLT PET,
- Substudy 2: 15 patients undergoing ^18^F-FDG PET, PK blood sampling and tumor
  biopsies,
- Substudy 3: 50 patients undergoing ^18^F-FDG PET and PK blood sampling.

**Fig. 1:**
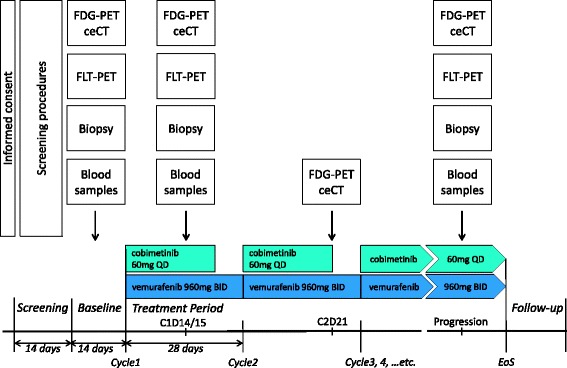
Study design scheme

Patients will be offered to participate in either one of the three substudies, depending on the wish and conditions of the patient. There is no randomization or blinding, all patients will receive the same treatment. Figure 1 shows a time schedule of the most important procedures.

### Study procedures

The schedule of study assessments and procedures are detailed in Table 2.

**Table 2:** Schedule of assessments and procedures

Period	Screening	Baseline study procedures			Vemurafenib/ Cobimetinib Treatment Period									Follow-up after treatment
Cycle	Within 28 days before start therapy	Within 14 days before start therapy	Cycle1			Cycle2		Cycle3	Cycle4	Cycle5	Cycle6	Cycle7+	At progression (EoS)	Every 12 weeks
Day			1	14	15	1	21	1 (± 3)	1 (± 3)	1 (± 3)	1 (± 3)	1 (± 3)	Within 7 days (on therapy)	
In- and exclusion criteria	**X**													
Medical history and demographics	**X**													
Interval medical history		**X**			**X**	X	**X**	X	X	X	X	X	**X**	X
Physical examination	**X**	**X**			**X**	X	**X**	X	X	X	X	X	**X**	X
Vital signs (Temp, HR, RR)		X			X		X	X	X	X	X	X	X	
Pregnancy test (if indicated)	**X**													
Dermatologic examination	X					X				X		X		X
Ophthalmologic examination	if indicated													
MUGA scan	X					X				X		X	X	
12-lead electrocardiography (ECG)	X	X			X	X	X	X		X		X		
Contrast Enhanced CT (CECT)	X	**X**			**X**		**X**		X		X	X	**X**	
ECOG performance status	**X**	**X**			**X**	X	**X**	X	X	X	X	X	**X**	X
Blood samples	**X**	**X**			**X**	X	**X**	X	X	X	X	X	**X**	
Informed consent	**X**													
PK blood sample		**X**			**X**								**X**	
DNA blood sample (Substudy 1 and 2)		**X**											**X**	
^18^F-FLT PET (Substudy 1)		**X**		**X**									**X**	
^18^F-FDG PET		**X**			**X**		**X**						**X**	
Tumor biopsy (Substudy 1 and 2)		**X**			**X**								X	
Adverse events		**X**			X	X	X	X	X	X	X	X	X	X
Start vemurafenib cobimetinib			X											

**X** *= Assessment/procedure performed to meet the objectives of the study,* X = *Assessment/procedure performed to monitor vemurafenib/cobimetinib treatment*

#### Screening and baseline period

After signing informed consent, patients will undergo screening procedures that include testing for the BRAFV600 mutation; laboratory tests (hematology, chemistries, LFTs); 12-lead ECG; left- ventricular function evaluation (MUGA), contrast-enhanced magnetic resonance imaging (MRI) or CT scan of the brain; contrast-enhanced CT of the chest, abdomen, and pelvis; and dermatologic assessments. After registration patients will undergo baseline procedures prior to the Treatment Period, including ^18^F-FDG PET, ^18^F-FLT PET (Substudy 1), PK blood sampling and tissue sampling (Substudy 1 and 2).

#### Study medication

During the Treatment Period vemurafenib will be taken orally at a starting dose of 960 mg BID, starting on Day 1 to Day 28 of each 28-day treatment cycle. Cobimetinib will be taken orally 60 mg once daily starting on Day 1 to Day 21 of each 28-day treatment cycle.

All patients will be closely monitored for safety and tolerability during all cycles of therapy, at the end-of-study treatment visit, and during the follow-up period. Treatment will continue until disease progression, death, unacceptable toxicity, or withdrawal of consent, whichever occurs earliest.

In patients with confirmed progressive disease according to RECIST v1.1 study treatment is discontinued when no benefit for the patient is expected anymore.

#### Response assessment

Tumor response will be evaluated with ceCT scans of neck, thorax, abdomen and pelvis according to RECIST v1.1. Any evaluable and measurable disease must be documented at screening and re-assessed at each subsequent tumor evaluation. The ceCT scans are performed at baseline, 2 weeks after start study medication, at the end of the seventh week, every 8 weeks thereafter and at progression following standard protocol. CT/MRI scans may be repeated at any time if progressive disease is suspected.

#### ^18^F-FDG and ^18^F-FLT PET imaging

In all 90 patients ^18^F-FDG PET scans are performed at baseline, Day 15 of Cycle 1, Day 21 of Cycle 2 and at progression. In 25 patients additional ^18^F-FLT PET scans are performed at baseline, Day 14 of Cycle 1 and at progression. Participating PET imaging sites must have the EARL FDG-PET/CT accreditation prior to the start of the study, and perform the PET scans to meet the standard requirements indicated in the European Association of Nuclear Medicine (EANM) guideline ‘FDG PET and PET/CT: EANM procedure guideline for tumour PET imaging: version 1.0’ [53]. All PET/CT scans are DICOM sent for central reviewing. All images will be assessed visually to identify sites of increased uptake. For quantification, a software package is used, which is developed for the specific purpose of quantifying PET images [54]. In all images, standardized uptake values (SUV) will be calculated for both ^18^F-FDG and ^18^F-FLT PET studies of sites showing the highest uptake. To evaluate and correlate both PET studies, but also to correlate the results of follow-up studies, a low-dose CT scan for attenuation correction will be used to make a head to head comparison between the uptake at different sites feasible.

#### Blood samples for pharmocokinetics

At baseline, Day 15 of Cycle 1 and at progression blood will be collected just before intake of the morning dose of vemurafenib and cobimetinib for pharmacokinetic purpose.

In a subset of patients treated in the Netherlands Cancer Institute (approximately *n* = 25), blood samples for a whole PK curve (pre-dose, 1 h, 2 h, 3 h, 4 h, 6 h and 8 h post-dose) will be collected on Day 15 of Cycle 1.

#### Blood samples for DNA sequencing

A peripheral blood sample is obligated for all patients who will undergo histological biopsy for DNA sequencing. Therefore, 1x10mL blood will be drawn at baseline and at the time of progression.

#### Tissue sampling for tumor characteristics

To evaluate the extent of inhibition of mutant BRAF, we will monitor the activity of the pathway in tumor tissue samples of 40 patients. Easily accessible progressive target lesions according to RECIST v1.1 will be biopsied before treatment, during treatment (*t* = 2 weeks) and at the time of proven progression. Tumor biopsies will be partly frozen or formalin fixated. Frozen tissue will be used for RNA extraction for RNA sequencing, genomic DNA extraction, next-generation sequencing and for phosphoproteomics, formalin fixed paraffin embedded material will be used for immunohistochemic analysis.

#### Monitoring adverse events

The NCI Common Terminology Criteria for Adverse Events (CTCAE) v4.0 will be used to characterize the toxicity profile of the study treatments on all patients. ECG assessments will be performed at screening, on Day 15 of Cycle 1, Day 1 and 21 of Cycle 2, Day 1 (± 3 days) of Cycle 3 and 5 and then on Day 1 (± 3 days) every 3 treatment cycles thereafter (Cycles 8, 11, 14, etc.). Dermatologic assessment will be performed at the beginning of Cycle 2 and then every 3 treatment cycles (± 3 days) thereafter (Cycles 5, 8, 11, etc.). Ophthalmologic examination is performed during treatment on suspicion of ocular toxicity. Left-ventricular function evaluation (MUGA) is evaluated throughout the study.

#### End of study and follow-up

The study will end when all patients enrolled have been followed for at least 3 years, withdrawal of consent, lost to follow-up, or the Sponsor decides to end the trial, whichever occurs first.

Patients may continue on study treatment until the development of progressive disease, unacceptable toxicity, and/or consent withdrawal. Patients who discontinue study treatment for any reason will be followed for development of squamous cell carcinoma, followed for disease progression and followed for survival until death, withdrawal of consent, or they are lost to follow-up.

### Statistical methods

#### Sample size calculation

Since ^18^F-FDG-PET scans are made at multiple time points (including baseline), the value of changes in metabolic activity can be studied in relation to overall response and time related endpoints.

There is a lack of evidence-based data in the assessment of therapy response and therapy resistance using ^18^F-FDG or ^18^F-FLT PET in patients with unresectable stage IIIc or metastatic melanoma treated with a BRAF inhibitor. Consequently, no robust power calculations can be performed to estimate samples sizes. However, given some feasibility aspects, some assumptions can be made about potential results and expected outcome. Based on recent publications [55, 56], it is expected that the PFS will be about 9 months. Assuming a hazard ratio of 0.5 or smaller and a 50–50 distribution in ^18^F-FDG PET positive and negative patients (either using the median or using the partial metabolic response of 25% reduction in the SUVmax) we can infer what median we would obtain.

Given the assumptions, the poor risk group would have a median PFS of 6.5 months and the good risk group a median of about 13 months. Assuming exponential survival, 18 months of accrual and another 18 months of follow-up (after the last patient has been enrolled), we would need to observe 66 events and 90 patients to enroll into the study (alpha 5% 2 sided and power 80%).

However, it should be stressed that these are assumptions and the nature of the study is explorative.

Experience with ^18^F-FLT is limited for monitoring response. Therefore, ^18^F-FLT will be ‘screened’ for its value by studying the association with ^18^F-FDG and CT in relation to response and outcome parameters. A review of studies looking at the value of PET in relation to response in various types of cancer, the average number of patients was 29 (data not shown, but available on request). The number of 25 patients is therefore considered both feasible and sufficiently valuable.

Important issues to solve in this series of patients are to develop a sensitive cut- off value for metabolic response (which may be different than the median or the cut-off for metabolic response found in other diseases), the association or potentially added value of the ^18^F-FLT PET-scan and the association with (high-dimensional) biomarkers.

Biopsies will be performed in a subset of 40 patients. This number is based on the paper of Dobbin and Simon 2007 in which sample sizes are calculated for building predictors [57]. The simulations indicated that with 2 differentially expressed genes, and 40 patients an effect size (2δ/σ) of 1.5 or more and with one differentially expressed gene, an effect size of 1.7 or better could be obtained.

#### Statistical analysis

Patient demographic data, tumor characteristics and data derived from the scans and biopsies will be described in frequency tables. In general, for continuous variables, mean, standard deviation, median, IQR and minimum and maximum will be given. Categorical variables will be presented with 95% confidence intervals whenever relevant (i.e. response).

SUV-parameters (mean, 3D peak, max) for ^18^F-FDG and ^18^F-FLT will be measured on a continuous scale and categorized or dichotomized using various rules available (PERCIST, and published disease specific rules). CT will be expressed according to RECIST v1.1. Linear-by-linear non-parametric analysis will be used for associations between ^18^F-FDG and ^18^F-FLT PET imaging and pharmacokinetics parameters and ceCT. Logistic regression and ROC-analysis may be applied to investigating the continuous parameters in association with response (CR + PR)/non response or clinical benefit (CR + PR + SD)/progression. Accuracy measures and best cut-off values will be calculated for each separate time point (Day 15 of Cycle 1 and Day 21 of Cycle 2 respectively). Whenever applicable and possible, baseline values (of ^18^F-FDG and ^18^F-FLT and pharmacokinetics parameters) will be included as separate (fixed) covariables.

A series of analysis will be performed to study PET imaging compared to ceCT, currently considered the ‘gold standard’. Agreement of test results will be evaluated using Bland- Altman plots and results will be expressed in terms of bias and precision measures with corresponding confidence limits. Both ceCT and PET data (i.e. baseline, decrease) will be associated with PFS and OS by means of survival model predictive accuracy analysis (cox regression) to obtain estimates of (time-dependent) sensitivity and specificity, and (time-dependent) receiver operating characteristic (ROC) curves [58–60]. Survival estimates will be plotted in Kaplan-Meier curves and tabulated with 95% confidence intervals at fixed time points and at its median (including 95% confidence interval).

Log-rank tests will be used for comparing different groups with respect to survival outcomes. Cox-proportional hazard analysis will be used to calculated hazard ratios and to model survival in the presence of potential imbalances or confounders.

## Discussion

In the majority of irresectable stage IIIc or metastatic BRAFV600 positive melanoma patients treated with combined BRAF/MEK inhibitors, metabolic alterations occur rapid after the initiation of therapy. Molecular imaging with PET visualizes metabolic activity in tumors and is a sensitive method to detect alterations in cell metabolism, even shortly after the start of therapy.

By detecting these metabolic alterations, responders to BRAF/MEK inhibition might be distinguished from non-responders at an earlier phase compared with anatomical imaging with ceCT and might predict resistance which occurs in almost all of these patients.

The REPOSIT study will enroll patients with unresectable locally advanced stage IIIc or stage IV melanoma as defined by the American Joint Committee on Cancer (AJCC) classification v.7. This study will be conducted only in patients whose melanoma harbors the BRAFV600E or BRAFV600 K mutation. Patients will be treated with vemurafenib plus cobimetinib and are monitored with PET/CT to assess the value of PET imaging in early response monitoring and resistance prediction. The information obtained from histopathological tissue characterization and pharmacokinetic analysis will be complementary to the imaging approaches; together they will allow us to draw a complete picture of early resistance mechanisms, aiming to make better predictions on the duration of response and development of resistance to treatment. We expect that the obtained results of this study will enable the development of personalized treatment selection strategies that in the future may select those patients who will have long term benefit from this treatment and prevent the initiation of an ineffective therapy and accompanying toxicity. Furthermore, this study may provide further insight in the mechanisms of resistance to combined BRAF and MEK inhibition.

## Abbreviations

(ce)CT: (contrast enhanced) computed tomography
^18^F-FDG: ^18^F-Fluorodeoxyglucose
^18^F-FLT: ^18^F-fluoro-3′-deoxy-3′-L-fluorothymidine
CR: Complete response
FDA: Food and drug administration
MAPK: Mitogen activated protein kinase
MRI: Magnetic resonance imaging
OS: Overall survival
PET: Positron emission tomography
PFS: Progression-free survival
PK: Pharmacokinetics
PR: Partial response
RECIST: Response evaluation criteria in solid tumors
SD: Stable disease
SUV: Standardized uptake value
